# Developing scientifically validated bias and diversity trainings that work: empowering agents of change to reduce bias, create inclusion, and promote equity

**DOI:** 10.1108/md-06-2021-0839

**Published:** 2022-09-05

**Authors:** William Taylor Laimaka Cox

**Affiliations:** Department of Psychology, University of Wisconsin–Madison, Madison, Wisconsin, USA; Inequity Agents of Change, Madison, Wisconsin, USA

**Keywords:** Training, Diversity, Equity, Bias, Unconscious bias, DEI

## Abstract

**Purpose –:**

Research consistently shows that non-scientific bias, equity, and diversity trainings do not work, and often make bias and diversity problems worse. Despite these widespread failures, there is considerable reason for hope that effective, meaningful DEI efforts can be developed. One approach in particular, the bias habit-breaking training, has 15 years of experimental evidence demonstrating its widespread effectiveness and efficacy.

**Design/methodology/approach –:**

This article discusses bias, diversity, equity, and inclusion (DEI) efforts from the author’s perspective as a scientist–practitioner – the author draws primarily on the scientific literature, but also integrates insights from practical experiences working in DEI. The author provides a roadmap for adapting effective, evidence-based approaches from other disciplines (e.g. cognitive-behavioral therapy) into the DEI context and reviews evidence related to the bias habit-breaking training as one prominent demonstration of a scientifically-validated approach that effects lasting, meaningful improvements on DEI issues within both individuals and institutions.

**Findings –:**

DEI trainings fail due to widespread adoption of the information deficit model, which is well-known as a highly ineffective approach. Empowerment-based approaches, in contrast, are highly promising for making meaningful, lasting changes in the DEI realm. Evidence indicates that the bias habit-breaking training is effective at empowering individuals as agents of change to reduce bias, create inclusion, and promote equity, both within themselves and the social contexts they inhabit.

**Originality/value –:**

In contrast to the considerable despair and pessimism around DEI efforts, the present analysis provides hope and optimism, and an empirically-validated path forward, to develop and test DEI approaches that empower individuals as agents of change.

The last several years have seen a renewed, energetic attention toward social justice issues related to race, gender, and other historically oppressed groups. As more people recognize the need to address individual and institutional forms of bias within various aspects of their lives, there has been an increase in requests for workplace trainings and interventions related to bias, diversity, equity, and inclusion (DEI). Correspondingly, DEI trainings have proliferated under a variety of labels (e.g. “Diversity Training,” “DEI training,” “Sensitivity Training,” “Cultural Competency Training,” “Anti-Racism Training”). For simplicity, this article will use the term “DEI training” to encompass all of these types of training programs meant to create positive changes related to bias, diversity, equity, and inclusion.

Choosing from the broad array of DEI trainings can be difficult for managers or human-resources departments considering a training for their workplace. How does one know a training is effective? How can they be sure it will teach concepts that are relevant and applicable in their particular industry? Will a training help accomplish company goals? Finding answers to these questions is not a simple task, and navigating these issues is often frustrating. When it exists, the systematic research on current DEI trainings is clear, and unfortunate: at best they do not work, and at worst they exacerbate bias and diversity problems (for excellent reviews, see al-Gharbi, 2020; Cox and Devine, 2019; Devine and Ash, 2021; Paluck *et al*., 2021; Pendry *et al*., 2007). Mainstream awareness of the issues with corporate and academic DEI trainings has increased, with major news outlets, including Scientific American, Forbes, Business Insider, TIME, and many others releasing articles denouncing that DEI trainings do not work (e.g. Green and Hagiwara, 2020; King, 2020; Lipman, 2018; Tang and Huang, 2021). This state of affairs is undoubtedly very frustrating for individuals or institutions interested in making meaningful changes related to bias and diversity. Is there any hope for those who want to reduce bias, create inclusion, and promote equity in their organizations?

In the present article, I argue that there is indeed hope – the abundant failures of the DEI industry arise from a root cause that is well-understood and has been identified and overcome in many content areas outside of DEI. As such, we can learn from efforts in other content areas how to develop approaches that create positive changes. Specifically, most DEI trainings adopt *information deficit model* approaches, which assume recipients lack key information, then try to correct that deficiency. Often used as an intuitive first attempt at creating change, the information deficit model’s shortcomings are well-documented, and there are many alternative approaches that more effectively create change (e.g. McDivitt, 2016; Suldovsky, 2017). In contrast to deficit models that treat people as passive recipients of information, *empowerment-based* approaches respect people’s autonomy and equip them to be effective, self-motivated agents of change within themselves and within their institutions (Cox and Devine, 2019). I discuss insights into how these kinds of approaches can address key challenges faced by DEI trainings, and, finally, review 15 years of experimental evidence demonstrating the efficacy of one particular empowerment-based DEI training, *the bias habit-breaking training*. In contrast to standard, non-scientific approaches that have proliferated in the public domain, the bias habit-breaking training has shown considerable promise at creating lasting, meaningful changes, at both individual and institutional levels (e.g. Cox and Devine, 2019; Cox *et al*., 2022; Devine *et al*., 2012, 2017; Forscher *et al*., 2017).

## Standard DEI trainings are just the latest failures of the information deficit model

The state of DEI trainings currently being implemented in the world, including their common content, approaches, and assessments of their efficacy (if any) has been extensively reviewed elsewhere. Rather than duplicating the superb work of other authors here, I will briefly state that the overwhelming consensus is that (1) DEI trainings are largely non-scientific and not experimentally tested, (2) the limited but consistent non-experimental evidence suggests that at worst they cause more problems for organizational climate, and at best they are ineffective at creating lasting, meaningful change, and (3) experimental tests of their common components lead to more negative outcomes than positive outcomes related to bias, diversity, inclusion, and equity (for excellent reviews, see al-Gharbi, 2020; Cox and Devine, 2019; Devine and Ash, 2021; Paluck *et al*., 2021; Pendry *et al*., 2007; see also Bezrukova *et al*., 2016; Brady *et al*., 2015; Brannon *et al*., 2018; Brewer *et al*., 1999; Cooley *et al*., 2019; Dobbin and Kalev, 2013, 2016; Dobbin *et al*., 2007, 2015; Dover *et al*., 2014; Duguid and Thomas-Hunt, 2015; FitzGerald *et al*., 2019; Frisby and O’Donohue, 2018; Kalev *et al*., 2006; Kaiser *et al*., 2013; Legault *et a*l., 2011; Naff and Kellough, 2007; Nordell, 2021; Wilton *et al*., 2019).

The lack of evidence in favor of DEI trainings, coupled with the abundance of evidence against them, has led many to directly question the ethics of implementing untested, non-scientific DEI trainings (e.g. al-Gharbi, 2020; Cox and Devine, 2019; Dobbin and Kalev, 2013, 2016; Nordell, 2017; Paluck, 2012). Nearly every major scientific organization (e.g. NIH, NSF, AAAS) has called for experimentally-tested, evidence-based approaches to addressing bias and promoting diversity (e.g. Moss-Racusin *et al*., 2014). Paluck powerfully argued that we should consider experimental testing of DEI trainings to be an “ethical imperative” (Paluck, 2012). DEI training is estimated to be an $8 billion industry (Lipman, 2018), and some have gone so far as to declare it a *failed* industry (Newkirk, 2019).

I argue that the pervasive failures of the DEI industry have close, fundamental ties to failures at creating change in other content areas. Specifically, DEI trainings overwhelmingly adopt an *information deficit model*, which has long been identified as a ubiquitous but highly ineffective approach to creating cognitive-behavioral change (Boykoff, 2011; Dickson, 2000; Irwin and Wynne, 1996; McDivitt, 2016; Miller, 2001; Schultz, 2002; Suldovsky, 2017). When seeking to change behavior in an audience (e.g. the lay public, students, employees), the first impulse of many communicators is to educate that audience – to give them information they are perceived to lack. The assumption inherent to this approach is that people fail to engage in desired behaviors (or persist in undesired behaviors) due to a knowledge deficit. Therefore, correcting that deficit is the apparent and obvious path to change (Boykoff, 2011; Dickson, 2000, 2005; McDivitt, 2016; Miller, 2001; Schultz, 2002; Suldovsky, 2017). A dermatologist, for example, might assume that people fail to wear sunscreen because they do not understand their risk and the dangers of skin cancer. The dermatologist then believes that teaching people about how sunscreen can help prevent skin cancer will correct the knowledge deficit and therefore be effective at getting people to wear sunscreen. Often, providing this information is the end of the dermatologist’s involvement, because their proximal goal – to correct the information deficit – has been met. When deficit model approaches like this are experimentally tested, however, the net effect is most often either no change or *decreases* in the desired cognitive or behavioral outcome.

Importantly, those employing deficit model approaches do not necessarily do so *consciously* (Suldovsky, 2017). Rather, the deficit model is a default approach because it is intuitively appealing and seems as though it should be effective – after all, if lack of information is the problem, then providing more information is the obvious solution. Communicators in or from academia may be especially prone to adopting these approaches, because a core tenet of the academic world is to generate, and then share, knowledge. The intuitive appeal of the deficit model has made it ubiquitous, even though its documented failures are legion (e.g. in the realms of smoking behaviors, McCarthy, 1985; Tønnesen, 2002; climate change McDivitt, 2016; Schultz, 2002; Suldovsky, 2017; sunscreen use, Jensen *et al*., 2020; Kantor, 2020; and vaccines, Dubé *et al*., 2013, 2015; Hornsey *et al*., 2018; Jarrett *et al*., 2015; Rossen *et al*., 2016; Sadaf *et al*., 2013; Smith, 2017; Trevors and Duffy, 2020). Despite widespread criticism, abundant evidence of its failure, and even being officially discredited by scientific bodies, the deficit model repeatedly resurfaces (Boykoff, 2011; Dickson, 2000; Kearnes *et al*., 2006; McDivitt, 2016; Miller, 2001; Suldovsky, 2017).

The clear trend of deficit model approaches is that they either do nothing or make problems worse – a trend echoed by DEI trainings. Efforts in the DEI context can benefit from lessons learned in other content areas, to move away from the default of the information deficit model and toward more effective, scientifically validated models of change. The information deficit model adopts a paternalistic, top-down approach that treats people as passive processors of information, and it tacitly assumes that behavior is determined by a single predictive factor: the presence or absence of relevant knowledge (Marteau *et al*., 1998; McDivitt, 2016; Miller, 2001; Simis *et al*., 2016). The stated or inferred foundation of these approaches is that there is something “wrong” with the recipients, which is predestined to make people feel defensive and to decrease motivation to change (i.e. it creates reactance; Brehm and Brehm, 2013). Further, people’s behavior is determined by a complex interplay of factors, including not only their knowledge, but their values, motivations, goals, sensitivity to social pressures, anxieties, and many other social and psychological forces (Boykoff, 2011; Cox, 2015; Dickson, 2000; Kearnes *et al*., 2006; McDivitt, 2016; Suldovsky, 2017; Sweeny *et al*., 2010; Wynne, 1992). Developing an intervention that effects meaningful, lasting changes in people’s behavior related to a given content area requires not only expertise in that content area, but expertise in intervention science and the science of cognitive-behavioral change.

To my knowledge, no prior scholar has explicitly named the information deficit model as the root cause of the widespread failure of the DEI industry, but it becomes readily apparent when reading reviews that comprehensively document common DEI training methods and activities (e.g. Carter *et al*., 2020; Devine and Ash, 2021; Paluck and Green, 2009; Paluck *et al*., 2021; Pendry *et al*., 2007). As reviewed extensively by Pendry *et al*. (2007), very common DEI training content involves educating people about historical intergroup disparities, the existence of biases/disparities in modern society or the workplace, implicit bias, White privilege (McIntosh, 1988), microaggressions (Lilienfeld, 2017), how it feels to be excluded/ discriminated against (Byrnes and Kiger, 1990; Stewart *et al*., 2003), or the benefits of diverse workforces. As in other contexts, the information deficit model is predictably ubiquitous in DEI. Common DEI trainings (1) assume bias and equity issues arise from deficits in employees’ knowledge of biases, racism, sexism, and other DEI issues, and (2) believe that providing information or experiences to ‘correct’ these deficits will lead to meaningful cognitive-behavioral change. Organizational DEI efforts are just the latest example of the insufficiency of the information deficit model to effect meaningful, lasting change.

## A shift in approach: empowering agents of change

A core flaw of deficit model approaches is that they fail to enlist recipients as active, autonomous agents in the change process. These approaches fail to recognize people’s inherent better nature and make the mistake of trying to impose change on recipients. Dickson (2000) previously argued that a clear remedy to deficit model approaches is to adopt empowerment-based approaches. The foundation of empowerment-based approaches is to center individuals as the primary drivers of their own change process (see Cox and Devine, 2019). Rather than change being something that is done *to* people, empowerment-based approaches focus on working *with* people, and empowering them as autonomous agents of change (see also Hennes *et al*., 2018). Contrasted with the deficit model’s notion that information is the sole determinant of behavior, empowerment-based approaches engage dynamically with people’s preexisting values, motivations, social connections, and other psychological processes. For an empowerment-based DEI approach, the starting point is to respect people’s autonomy and believe that they have good intentions with regard to bias and diversity issues (Cox and Devine, 2019). Although empowerment-based approaches may teach people new information, this education is framed as *enhancing* people’s existing knowledge and experiences, rather than correcting a deficit.

This shift from deficit to empowerment approaches has parallels in many other domains that have recognized the benefit of shifting from paternalism to autonomy (Cook, 2001). In social and behavioral sciences, a strong parallel is the paradigm shift from treating and calling people “subjects” of the research to considering people to be willing and informed “participants” (e.g. Boynton, 1998; Sales and Folkman, 2000). Recent medical scholars have made the case for transitioning away from the traditional medical model, in which medical professionals give directives that patients are expected to obey, to models like patient-centered care, in which medical professionals strive to give patients the tools to be informed decision-makers and then work *with* the patients to develop treatment plans (e.g. Kumar and Chattu, 2018). Similar discussions in management science have arisen in recent years, emphasizing transformational leadership over transactional and directive leadership styles (Arnold and Loughlin, 2013; Odumeru and Ogbonna, 2013), or employee-centered/human-centered management as opposed to mechanistic management (Baker *et al*., 1996; Hoogervorst *et al*., 2005). In different contexts, these and other recent conversations have all emphasized the benefits of moving away from paternalistic approaches that tell people what to do and toward approaches that respect people’s autonomy and give them tools to direct their own actions toward shared goals (see also Howe *et al*., 2022).

The success of an empowerment-based approach requires that people’s autonomous, intrinsic values and motivations are compatible with the overarching goals of the training. Fortuitously, basic research has long demonstrated considerable reasons for optimism about trusting people’s autonomy around DEI topics. Most people hold strong personal values that oppose bias, prejudice, and inequity – they want to treat others fairly, unencumbered by biases or prejudgments (e.g. Cox, 2015; Devine, 1989; Devine *et al*., 1991; Monteith, 1993; Monteith *et al*., 2001, 2002; Plant and Devine, 1998, 2009). Since the 1980s, research has shown repeatedly that the average person believes in fairness, and will become motivated to reduce bias and inequity if they are made aware that they may, however unwittingly, be vulnerable to intergroup biases (Amodio *et al*., 2007; Cox, 2015; Devine, 1989; Devine *et al*., 1991; Monteith, 1993; Monteith *et al*., 2001, 2002; Plant and Devine, 1998, 2009) [1].

Motivation to address bias and inequity, however, is not sufficient – very often, people direct their motivation in unhelpful directions, adopting intuitive tactics that seem like they *should* help reduce bias but that actually backfire, or are simply inadequate (e.g. Apfelbaum *et al*., 2008, 2012; Kulick *et al*., 2000; Norton *et al*., 2006; Uhlmann and Cohen, 2007). At the individual level, for example, trying to suppress stereotypes leads a rebound effect where people display *more* bias, rather than less (Macrae *et al*., 1994). At an institutional level, implementing trainings that use the information deficit model, as discussed above, likewise backfire. The focus of an empowerment-based approach, therefore, is to engage people’s preexisting personal values and motivations related to DEI, guide them away from common ineffective tactics, and help them to autonomously direct their efforts in more effective directions. To identify effective *methods* to guide the change process in this way, one can look to other areas of work that have developed and successfully implemented models of self-sustaining cognitive-behavioral change.

## Scientific methods to effect lasting cognitive-behavioral change

Any effort to effect meaningful changes in people, whether related to bias and diversity or any other context, should make use of the vast literature on the science of cognitive-behavioral change. In fact, many scientists have long argued that, rather than treating intergroup biases as a “special” or fundamentally distinct type of psychological phenomena, they should instead be understood as arising from ordinary psychological processes (Allport, 1954; Bodenhausen and Macrae, 1998; Cox and Devine, 2015; Cox *et al*., 2012; Devine, 1989; Fiske, 1998). The social *impact* of biases may be special, but their underlying cognitive origins are not. Efforts to create lasting, meaningful cognitive-behavioral change related to DEI, therefore, can build on effective approaches from other content areas.

### The habit model

Devine (1989) has long argued that intergroup biases can be likened to habits of mind. From the media and their social environments, kids learn stereotypes about major social groups and display biases based on those stereotypes at ages as young as 4, 5, and 6 years old (Bigler and Liben, 2007; Levy and Killen, 2008; Pauker *et al*., 2010). These stereotypes and biases are reinforced over the lifetime, making them automatic, default responses that are often at odds with conscious values and intentions – just like bad habits (Devine, 1989). As adults, we have the cognitive reasoning capacity to regulate these habitual stereotypes and biases, but we continue to be bombarded by stereotypes in the media, and many processes in our cognitive systems (e.g. confirmation bias; illusory correlation) help perpetuate these stereotypes in our minds (Chapman, 1967; Cox *et al*., 2012; Cox *et al*., 2022; Darley and Gross, 2004; Gibson *et al*., 2013; Kalish *et al*., 2011; Nickerson, 1998; Pohl, 2004). Biases, like other habits, are automatic, persistent, and often operate at odds to conscious intentions. If we consider biases as habits of mind, one potentially fruitful approach to changing biases is to draw on the habit change literature (e.g. see Wood, 2017; Wood and Neal, 2016) and to approach bias reduction as a process of breaking a habit (Cox and Devine, 2019; Devine, 1989; Devine et al., 2012).

Considering the notion of biases as habitual highlights a major way empowerment-based approaches can succeed where the information deficit model fails. A core flaw of the deficit model is the tacit assumption that persistence of undesired behaviors (or lack of desired behaviors) occurs due to a *passive* lack of information. Habits, however, are maintained by interacting, *active* forces for inertia (e.g. Cox *et al*., 2022; Reggev *et al*., 2021). Combatting this inertia requires active, sustained effort over time. Empowerment-based approaches are well-suited to imparting this message, if they give people *actionable* tools to employ in the service of combatting biases and help make the change process *self-sustaining* over time. Approaching bias and inequity reduction using a “habit” model is especially useful for addressing this challenge, because people intuitively understand that habits have considerable inertia, and inherent to the notion of breaking a habit is that it requires sustained effort over time (Cox and Devine, 2019).

### Cognitive behavioral therapy

In many ways, stereotypes and biases are directly parallel to other types of “habits of mind”, especially negative self-schemas that have been of interest to clinical researchers (Cox *et al*., 2012). Just as stereotypes are automatically activated cognitions that negatively affect judgments, feelings, and behaviors toward others, negative self-schemas in depression are automatically activated cognitions that negatively affect judgments, feelings, and behaviors toward the self. Both intergroup stereotypes and negative self-schemas are well-learned, well-rehearsed cognitive structures that are automatically activated, are difficult to change, can bias attention and information processing, and have an array of cognitive, affective, and behavioral consequences that are often at odds with conscious intentions (see Cox *et al*., 2012 for a more comprehensive review; Bargh and Tota, 1988; Beck, 1967; Beck and Alford, 2009; Devine, 1989; Dunn and Spellman, 2003; Eaves and Rush, 1984; Fiske, 1998; Hamilton and Trolier, 1986; Hilton and von Hippel, 1996; Wenzlaff *et al*., 1988). Building on this insight, Cox *et al*. (2012) argued that methods from experimental clinical research, especially cognitive-behavioral therapy (CBT), could be useful for DEI efforts (see also Beck, 1999).

CBT is one of the oldest and most widely-applied behavioral change frameworks, and it has been extensively validated in decades of experimental studies (for reviews, see Cuijpers *et al*., 2016; Hofmann *et al*., 2012, 2013). CBT enlists the participant (or patient) as an active agent of their own change, helping them to identify maladaptive cognitions and behaviors, teaching them to understand the impact of those cognitions and behaviors, and giving them concrete cognitive and behavioral tools to help them change the maladaptive cognitions/behaviors. CBT for depression, for example, is as efficacious as medication for depression, and also reduces likelihood of relapse after treatment ends, because CBT equips people to continue their therapeutic work themselves (Beck, 2005, 2021; Hollon, 2003; Hollon and Dimidjian, 2009; Hollon and Shelton, 2001). In the clinical realm, CBT is, by far, the most effective method at creating long-term change in individuals’ cognitive-behavioral processes and their affective consequences (Beck, 2005, 2021; Hollon, 2003; Hollon and Beck, 2013; Hollon and Dimidjian, 2009; Hollon and Shelton, 2001). CBT has been extensively studied and carefully refined since its origination in 1970s, and therefore has precise, established methods and parameters for effectively guiding long-lasting cognitive-behavioral change (e.g. Beck, 2005; Beck, 2021; Cuijpers *et al*., 2016; Hofmann *et al*., 2012, 2013). Some work even shows long-lasting beneficial effects from just a single session (e.g. Schleider and Weisz, 2018), or from CBT delivered via a computer application (e.g. Carroll *et al*., 2008; Cavanagh *et al*., 2006; Ebert *et al*., 2015; Himle *et al*., 2006; Luo *et al.,* 2020). CBT is also a very adaptable framework that can incorporate aspects of other approaches (e.g. mindfulness therapy, motivational interviewing) as needed or as appropriate. Crucial to CBT is that clients are taught how to recognize, manage, and address automatic cognitions and behaviors autonomously – CBT gives them tools to make their change process self-sustaining over time (Beck, 2021). Cox *et al*. (2012) developed an integrated theoretical model adapting CBT methods as a self-sustaining approach to reduce bias. Drawing on well-established, effective methods for cognitive-behavioral change provides a clear blueprint for guiding change in a DEI context.

The versatility of CBT is especially useful for DEI efforts, given that there are infinite forms of diversity, and therefore bias, to consider. People are vulnerable to showing biases related to race, gender, sexual orientation, political orientation, religion, disability, age, many other social identities, and the intersections thereof (Cox *et al*., 2012; Crenshaw, 1989). It is infeasible for organizations to implement separate interventions or training programs for every stereotyped group that may be affected by biases/disparities (e.g. Black people, Latin people, Asian people, Muslim people, women, LGBT + people, people with disabilities, and their intersections). In addition to the diversity of potential target groups, bias and inequity take diverse forms, across and within individual people, time periods, circumstances, and institutions (Cox *et al*., 2012). Considering the various target groups and forms they take, biases and inequities have truly limitless variability. No training approach could hope to comprehensively tally them all.

Considering the infinite variability of biases highlights another crucial way that information deficit model approaches fail. Because the deficit model relies on directive information transfer, audience learning is largely limited to, at most, a “laundry list” of facts, which, in the case of biases/inequities, will inevitably be incomplete. Empowerment-based approaches, on the other hand, teach skills that enable recipients to autonomously recognize and address novel issues that may arise. Insights from CBT are especially relevant to addressing this challenge: A therapist cannot anticipate all the specific forms their client’s myriad stressors and life circumstances will take in the future, so the therapist must teach the client *generalizable* and *customizable* skills (Beck, 2021). A DEI training must likewise teach generalizable and customizable skills, so recipients can identify and address the infinite variability of biases and inequities they may encounter. By centering the individual, not the trainer, as the primary agent of change, empowerment-based approaches provide a method to address the infinite varieties of bias and inequity.

## The bias habit-breaking training: empowering agents of change

One empowerment-based approach that encapsulates the principles, methods, and challenges reviewed above is the bias habit-breaking training (also sometimes known as the “prejudice habit-breaking intervention” or the “break the bias habit” workshop; Carnes *et al*., 2015; Cox *et al*., 2022; Devine *et al*., 2012; for another review see Cox and Devine, 2019). Since 2007, this training was developed as an approach to empower people to be autonomous agents of change. In this context, *agents of change* are defined as individuals who are self-motivated and actively engaged in reducing bias, creating inclusion, and promoting equity, both within themselves and within the institutions and social systems they interact with. The training is designed to help them sustain these efforts across their lifetimes, equipping them to seek out and address new forms of bias whenever and wherever they arise.

The bias habit-breaking was developed, experimentally tested, and iteratively refined and updated over the past 15 years. Common across all versions of this approach is that participants are taught Devine’s (1989) prejudice habit model, which discusses biases and stereotypes as “habits of mind” and describes the process of overcoming these biases as breaking a habit (Devine, 1989). Breaking a habit is an intuitive, familiar idea for people, making it easy to apply in the context of addressing biases. Core to the habit-breaking approach is that breaking a habit requires ongoing effort over time, setting people up to understand that they must make their efforts self-sustaining.

Of key interest is relaying the intervention content in a way that will maximize recipients’ retention of the information, motivation to work on DEI issues, and likelihood of sustaining these efforts over time. Building on Cox *et al*. (2012) framework, the latest versions of the bias habit-breaking training adapt core principles from CBT to help meet these goals (Beck, 2021; see Table 1).

The intervention content teaches people how and *why* biases occur, encouraging them to autonomously seek out and identify the variable forms of bias that may occur in their own lives, and to self-sustain this process over time. It also teaches them actionable, generalizable, customizable tools that they can use, if they so choose, to reduce bias, create inclusion, and promote equity, related to any target group. There are a number of tools, including tools that involve retraining cognitive/emotional reactions to reduce bias, procedures that can be put into place to prevent bias, ways to create more inclusive environments, how to effectively speak up about bias, and other topics. Integral to the training is that biases and inequity are discussed in a very evidence-based, non-accusatory way – it frames the scientific evidence as demonstrating people’s unwitting *vulnerability* to biases, rather than presenting bias as inevitable or indicative of someone’s moral character. Each component of the training is carefully crafted to directly address challenges created by biases, and to help recipients be motivated, effective, autonomous agents of change.

### Evidence of effectiveness and efficacy

The present article has drawn on both my expertise as a cognitive-behavioral scientist and my applied experience as a DEI practitioner. As a scientist, I rely on systematic, experimental data for my confidence that the bias habit-breaking training has beneficial, long-lasting effects. As a practitioner, I also have the privilege of witnessing firsthand how attendees engage with the training and apply it. I draw on both kinds of evidence in the following sections: In addition to reviewing published and ongoing quantitative, empirical research testing the bias habit-breaking training, I will also share a few (admittedly anecdotal) examples of real-world impacts the training has had on individuals and institutions.

Several experimental studies have demonstrated lasting and impactful effects of the bias habit-breaking training. As noted earlier, the training has been iteratively tested, refined, and updated over the last 15 years. The very early versions of the intervention focused more narrowly on one type of intergroup bias (e.g. race bias, Devine *et al*., 2012; Forscher *et al*., 2017; gender bias, Carnes *et al*., 2015). Following the initial success of these early versions, the training was updated to cover *general* principles of how biases can play out related to any sort of target group and in any situation or context (e.g. Cox *et al*., 2022).

Following several randomized-controlled experiments demonstrating the effectiveness of the bias habit-breaking training, my colleagues and I began receiving requests to deliver the training live, independent of any formal empirical study. Given the evidence we had amassed at that point, we felt confident transitioning from testing to practical application, and we have delivered the training live for many companies, organizations, academic institutions, and governmental agencies around the world. Through these practical endeavors, we have been able to witness firsthand how the bias habit-breaking training impacts people and organizations. Anecdotally, I can say that nearly every time the training is delivered, someone in the audience has what my colleagues and I call an “Aha! Moment”, where they discover some way that they have been expressing bias or contributing to inequity without realizing it. Very often, these are highly specific forms of bias or inequity that are particular to the individual or a particular workplace – examples unlikely to be captured in typical empirical, quantitative studies. In the following sections, after reviewing the empirical findings, I share a selection of these firsthand examples, to showcase the breadth of potential outcomes that can be influenced when individuals are situated and empowered as the primary agents of change.

I organize the review of these empirical findings and firsthand examples under three broad themes that correspond to key areas where DEI programs seek to make progress: Reducing Bias, Creating Inclusion, and Promoting Equity.

#### Reducing bias.

In the very first test (Devine *et al*., 2012) of the first version of the habit-breaking training, 91 undergraduate students completed an array of baseline measures, then were randomly assigned to serve as controls or to receive the training. They completed follow-up assessments at several timepoints, up to 8 weeks post-manipulation. The first outcomes of interest were participants’ self-reported levels of awareness of their vulnerability to express bias unintentionally and their concern that racial bias was a serious problem (see also Carter *et al*., 2020 for discussion of these constructs’ importance). Whereas control participants’ scores on these measures remained unchanged over time, training participants’ scores significantly increased, indicating the training was effective at making people more aware of their potential to express race bias unintentionally and more concerned that race bias was a serious problem. These effects were later replicated in two other, higher-powered randomized controlled trials (Cox *et al*., 2022; Forscher *et al*., 2017).

In the initial randomized-controlled trial (Devine *et al*., 2012), training participants’ levels of implicit bias, as measured by the Implicit Association Test (IAT), significantly decreased, whereas control participants’ IAT scores did not. This effect endured to the end of the study, 8 weeks post-manipulation. This effect was unprecedented in the literature at the time, and it remains so today. In a recent meta-analysis of 492 experiments trying to reduce implicit bias (Forscher *et al*., 2019), no study demonstrated decreases in implicit bias that lasted more than 24 h, with most lasting only a few minutes (see also Lai *et al*., 2016; Siden *et al*., 2022). Work on the bias habit-breaking training is the sole exception to these patterns.

In another study, 302 undergraduate participants were randomly assigned to receive the bias habit-breaking training or to serve as controls (Forscher *et al*., 2017). These participants completed IATs every other day for two weeks, in an attempt to model the time-course of the previously observed reduction in IAT bias. Replicating Devine *et al*. (2012) pattern, the training participants’ IAT scores decreased over time. Contrasting with the prior study, however, the control participants’ scores also decreased. Given that the participants completed the IAT with such a high frequency (up to 8 times in a two-week period), it seemed that this pattern constituted a practice effect on the IAT task. This interpretation matches other studies that show learning effects in the IAT task (e.g. Cochrane *et al*., 2022). Devine *et al*. (2012) original pattern of reduced implicit bias only in the training condition has recently been replicated in a new randomized-controlled experiment with a much larger sample size (*N* = 957). In this replication (Cox *et al*., 2022), participants completed the IAT two weeks and six weeks after random assignment, and training participants again significantly decreased in implicit bias, whereas control participants’ IAT scores remained unchanged. This effect was observed up to the latest timepoint during which IAT scores were collected, at 6 weeks post-manipulation. This pattern further supports the interpretation that the Forscher *et al*. data reflects a practice effect on the IAT, whereas the other two studies’ reductions in the training, but not control, conditions are more likely due to training participants implementing the bias reduction tools taught in the training.

The importance of a reduction in measured implicit bias lies in the assumption that this reduction will correspond to reductions in other outcomes; the IAT is very often used as a proxy for discriminatory judgments and behaviors (Cox and Devine, 2022). In the Forscher *et al*. (2019) meta-analysis, when studies assessed implicit bias interventions alongside behavioral outcomes, observed decreases in IAT scores did not mediate corresponding reductions in biased behavior. This lack of mediation indicates that those reductions in measured implicit bias are unlikely to be meaningful for other outcomes (Forscher *et al*., 2019). Again, work with the bias habit-breaking training appears to be the sole exception. A subsample (*N* = 320) of Cox *et al*. (2022) participants were recruited for an ostensibly unrelated study 2–3 years after being randomly assigned to receive the bias habit-breaking training or to serve as controls. These participants completed Monteith *et al*. (2002) stereotype regulation task. This measure is commonly used as an indicator of the amount of effort people will put into avoiding stereotypic assumptions (see Burns *et al*., 2017; Czopp *et al*., 2006). Training participants stereotyped less than controls on this task, and this effect was significantly mediated by the prior observed decrease in implicit bias (Cox *et al*., 2022). Unlike other documented reductions in implicit bias (Forscher *et al*., 2019), the reductions resulting from the bias habit-breaking training appear to be both long-lasting and related to other, meaningful outcomes.

Initial experimental tests of the training involved solely or mostly White participants, either by design (Devine *et al*., 2012; Forscher *et al*., 2017) or because the sample merely reflected the demographics of the predominantly White study population (Cox *et al*., 2022). In the interest of focusing on Black voices in this line of research, Auxier and Cox (2022) specifically recruited Black participants to complete the training and to share their perspectives and opinions about it, via an evaluation survey (*N* = 28) immediately after the training, and a qualitative interview with a Black interviewer (*N* = 12) at least two weeks after the training. One key concern was that, due to their frequent experiences as targets of bias, Black participants might see the training as irrelevant to their own cognitions and behavior. Contrary to this expectation, all 12 participants who completed interviews reported that they had applied what they learned in the training, either by directly using one of the bias-reducing tools, or by applying the content in a way that improved interactions with others. All 12 interviewees also reported that the training was respectful and appropriate in its treatments of biases and other issues related to Black people. Mirroring the increase in awareness observed in the initial studies with predominantly White participants, several of the interviewees reported that the training helped them tune in to forms of bias they might themselves express toward other social groups.

These patterns with Auxier and Cox’s participants are reflected in many of my firsthand experiences delivering the training. Many attendees who are members of historically disadvantaged groups (e.g. people of color, women, LGBT + people) have reported realizing that they may have disregarded their potential to express biases based on other group statuses, perhaps due to predominant social rhetoric that frames biases as arising solely or primarily from members of historically powerful groups. Because the training discusses bias as arising from ordinary cognitive processes that occur within everyone – not just White people, or men, or straight people, or people from other non-stigmatized social groups – it empowers everyone to look for and notice biases they might be vulnerable to expressing.

Indeed, attendees often realize, either in-the-moment or after the training, various ways they have been expressing biases without realizing it, and then make a plan to stop that bias. A teacher in one of our trainings was mortified when she realized, in-the-moment that, for years, she had been making stereotypic assumptions that disadvantaged her Black and Latin students. Specifically, if a Black or Latin student failed to turn in an assignment or came late to class, she would make the snap judgment that they were lazy and did not care about their coursework. When White students engaged in similar behavior, however, she more often gave them the benefit of the doubt and gave them opportunities to explain themselves. During another training, an attendee received a message from an employee saying he was going to be late to work that day. The attendee began getting angry with the employee, jumping to conclusions about him not caring about his job. This circumstance unfolded during the section of the training in which I explain tools that help people retrain their reactions to curb snap judgments. She applied one of these tools (*considering situational explanations for behavior*; e.g. Stewart *et al*., 2010) while I was discussing it, and immediately began to feel more calm, as her generated situational explanations for the employee’s behavior (e.g. “maybe his car broke down,” “maybe his childcare fell through”) gave her more compassion for him. She told me about the experience afterward via email and shared that her relationship with that employee had noticeably improved. Many more firsthand examples abound, and they reinforce the idea that some forms of bias are highly common across many people, and some are more specific or unique to a particular person or circumstance. Because the bias habit-breaking training puts people on a path to identify and correct biases themselves, it is effective at equipping them to identify and address bias in its myriad forms.

#### Creating inclusion.

In addition to reducing bias, a common goal of DEI initiatives is to encourage behaviors that create more inclusive environments. One important way that people engage in inclusivity is to speak up and confront biased or offensive rhetoric. Testing the training’s effects on speaking up, Forscher *et al*. (2017) followed up with their participants via an ostensibly unrelated study 1–2 years post-manipulation. Training and control participants received an email that they believed was sent by their university newspaper. The email asked them to be part of a program in the newspaper that allowed a student to write an editorial about a topic of their choosing, which was then reviewed by fellow students who could, if they so chose, write a reply that would be published alongside the editorial. The (fabricated) editorial participants read was titled “Racial stereotypes are useful tools”, and it argued that stereotypes are useful, harmless, and that stereotyping has only become “untrendy” because society is too politically correct. Participants (*N* = 79) rated their agreement/disagreement with the author’s perspective, and they were given the option to write a response to the editorial, which they believed could be selected to be published under their name in the school paper. Training and control participants disagreed with the authors’ perspective to the same extent, but training participants were 64% more likely to translate that disagreement into behavior, taking an ostensibly public stand against the rhetoric by writing a response to the editorial that contradicted its authors’ perspective (Forscher *et al*., 2017). These patterns provide further evidence for the value of an empowerment-based approach: the parity in training and control participants’s disagreement with the bias rhetoric demonstrated again that most people’s values oppose bias. The fact that training participants were more likely to speak up demonstrated that this training approach empowered them to translate those preexisting values into meaningful action.

Cox *et al*. (2022) likewise sought to examine speaking up about bias and inclusion related topics. A subsample (*N* = 304) of their replication study described above completed an ostensibly unrelated study 1–2 years post-manipulation. This study involved a mock online classroom discussion, where students discussed popular press articles related to “hot topics” in contemporary discourse. Two articles touched on bias/inclusion-related issues, one discussing Muslim people being targeted for extra security screenings at airports, and one discussing the need for gender nonbinary bathrooms. Importantly, these two DEI topics were not mentioned in the content of the training that the training participants had completed prior, thus enabling Cox *et al*. to examine whether training participants would generalize what they had learned beyond biases explicitly mentioned in the training. Quantitative text analyses assessed how much participants brought up bias and inclusion topics in their discussion of the Muslim and gender nonbinary articles. Compared to controls, training participants spoke up about bias/inclusion 181% more than control participants overall. For the Muslim-related article specifically, training participants discussed bias/inclusion topics 20.7% more than controls, and for the gender-nonbinary-related article, training participants discussed bias/inclusion topics 12.5% more than controls. Each of these effects was statistically significant. This pattern is consistent with the prediction that this empowerment-based approach would give participants tools to generalize and customize what they learned in the training to additional, novel forms of bias not addressed directly within the training.

Another component of creating an inclusive social climate is to help others to be more inclusive. In response to open-ended questions about their experience with the training, many participants report sharing what they learned with their peers, to help more people create inclusive environments (Auxier and Cox, 2022; Cox *et al*., 2022; Devine *et al*., 2012; Forscher *et al*., 2017). In both lab studies and real-world experimental implementation of the bias habit-breaking training, Forscher (2017) demonstrated that people explicitly taught others how to reduce bias and create inclusion. In one study, the effects of the training were even stronger on people who did not attend the training, but who worked closely with someone who did attend the training (Forscher, 2017). This effect may suggest that people not only share what they learned, but that as they do, they may customize the content even further, to have stronger effects in their specific context.

Organizational climate is another important indicator of creating inclusion. In another large-scale study, 92 academic departments in science, technology, engineering, and math (STEM) fields were randomly assigned to receive the bias habit-breaking training or to serve as controls (Carnes *et al*., 2015; Devine *et al*., 2017). This version of the training focused specifically on gender bias in STEM. Training faculty increased compared to controls on a number of self-report measures related to departmental climate, such as self-efficacy to address gender bias in their department, having taken action to promote gender equity, and awareness of bias. Carnes *et al*. (2015) linked their study data with an office on campus that annually collects faculty climate data, to assess whether climate changed in training versus control departments. Whereas control department climate metrics remained the same over time, training department climate significantly improved over time, with both women and men in training departments reporting better departmental “fit” and that their work was more respected by their colleagues (Carnes *et al*., 2015).

Several studies on the bias habit-breaking training have shown evidence of creating inclusion in the form of increased or improved intergroup interactions. In free response data, training participants often report seeking out or being open to more interactions with members of other groups, at rates higher than those of control participants (Cox *et al*., 2022; Devine *et al*., 2012; Forscher *et al*., 2017). In Auxier and Cox’s (2022) exploration of Black participants’ perspectives on the training, 9 of the 12 interviewees reported that the training changed how they interpreted other people’s potentially biased behaviors, leading them to be more understanding when someone else expresses bias unintentionally. In an ongoing study in Toronto, preliminary data indicate that doctors who were randomly assigned to receive the bias habit-breaking training received higher satisfaction marks from their Indigenous patients compared to doctors who were randomly assigned to serve as controls.

An attendee wrote me a year after he attended a session of the bias habit-breaking training to tell me about how the training changed his actions in a way that made a difference for his whole neighborhood. A few months after he attended the training, a Mexican family moved into his predominantly White neighborhood. His initial thought was that he and his family should not go introduce themselves to the new family, because, “They do not want some White people coming to bother them!”. But then he remembered part of the training that explicitly warned against making assumptions that people from different backgrounds will lack interest in having interactions (cf. “pluralistic ignorance”, Shelton and Richeson, 2005). He overcame his hesitance and took his family to welcome their new neighbors. Not only did this attendee and his family become close friends with their new neighbors, but their inclusive gesture inspired others in the neighborhood to overcome their own apprehensions, and, in this attendee’s words, it “set off a chain reaction” of welcoming and outreach.

#### Promoting equity.

**Figure 1.**
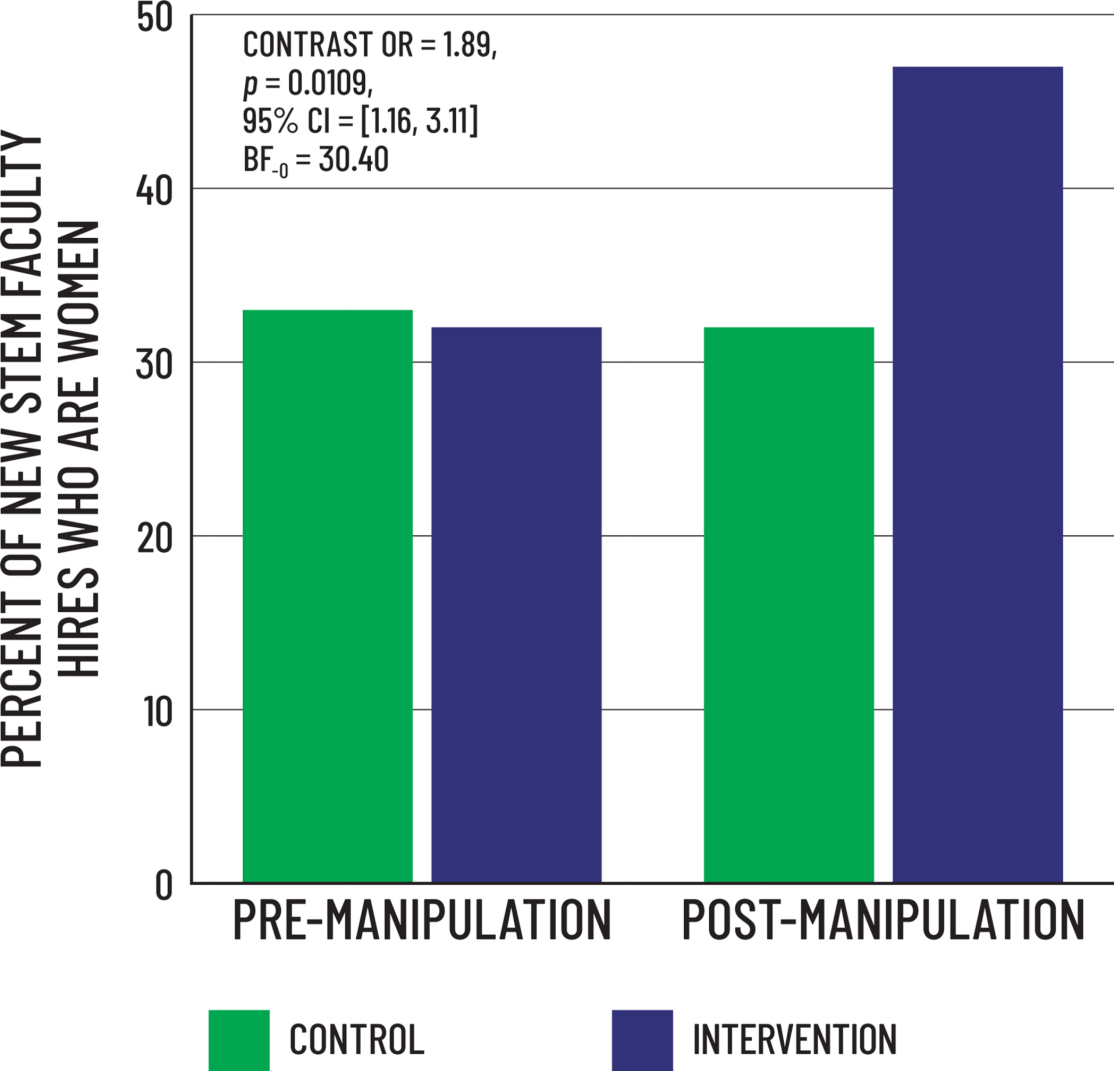
Hiring data - percent of new tenure-track faculty hires who were women

Inequity can take many forms, in different contexts. In academic STEM contexts, a crucial issue is inequity in the hiring of women as tenure-track faculty. A recent, long-term follow-up to the Carnes *et al*. (2015) study examined whether the training led to changes in hiring of women as new faculty (Devine *et al*., 2017; Forscher, 2017). Working with human resources data, observed hiring patterns during the two years prior to random assignment revealed that new tenure-track hires in both intervention and control departments were only 32–33% women. In the two years after departments either received the training or were randomly assigned to be controls, new hires in control departments were still only 32% women, but new hires in intervention departments were 47% women, Contrast OR = 1.89, *p* = 0.0109, BF_-0_ = 30.40. See Figure 1. In other words, the bias habit-breaking training caused a 43% increase in hiring of members of underrepresented groups.

These general hiring data were previously explored in Devine *et al*. (2017), but these analyses and figure are new for the present publication. The bar graph shows raw population percentages of women hired as new faculty during the 2-year periods before and after random assignment to intervention or control departments, as reported in Carnes *et al*. (2015). We conducted a Generalized Linear Mixed Effects Model set up as reported in Devine *et al*. (2017), except that we tested a contrast comparing hires in intervention departments post-manipulation to hires in control departments pre- and post-manipulation and intervention departments pre-manipulation. The proportion of women hired by intervention departments in the two years after the training was significantly higher than intervention departments in the two years before the training, or the control departments two years before or two years after the manipulation, OR = 1.89, *p* = 0.0109, 95% CI = [1.16, 3.11]. A Bayesian Independent Samples *T*-test (BF_-0_ = 30.40) for this contrast further indicated these data constitute *very strong* evidence that the bias training caused increased hiring of women.

Other, ongoing work is examining additional outcomes related to important equity indicators in different contexts. An ongoing study with middle and high school teachers and students in a large Californian school district is examining whether teachers completing the habit-breaking training has beneficial effects on achievement gaps among their students. Preliminary data with 176 teachers and 7,084 students indicate that when students have few teachers who received the habit-breaking training, there is a typical achievement gap, with Latin students having lower grades than White students. When students have a high proportion of teachers who received the training, however, that achievement gap disappears, driven by Latin students receiving higher grades (Saad *et al*., *in progress*).

Inequity becomes integrated into social systems and institutions in myriad forms, many of which are unique to specific organizations. This uniqueness makes it crucial that DEI efforts center on individuals within an organization as the agents of change (as in empowerment-based approaches), rather than the trainer as the driver of change (as in information deficit model approaches), because those *in* the day-to-day culture of an organization are the ones best equipped to identify and correct their organization’s particular inequities. At one company that received the bias habit-breaking training, learning about how inequities persist within organizational structures led an employee to investigate the method used by their company’s automatic mail-sorting machines, which stamped mail with an “L”, or an “H” based on ZIP code. The current mailroom employees had no knowledge of why the mail sorting machine did this. It had been programmed to follow the manual sorting procedures of the previous mailroom staff, who had done what they were taught from the generation before, and so on. The account managers who received the sorted mail knew that “L” and “H” indicated low and high priority, respectively, but they did not know how those determinations were made (one account manager thought the priority status was determined by artificial intelligence software identifying known high-value clients). The manual sorting procedure was revealed to have been a holdover from the 1960s, when the company was explicitly discouraging its employees from working with Black clients – the mail marked as low priority came from the zip codes of historically Black neighborhoods. No one currently at the company knew the explicitly racist origins of these procedures, and they were, rightly, appalled when they were brought to light. This instance of institutional inequity only came to light because one employee took it upon himself to act as an agent of change to investigate and correct the problem.

During a training at a large financial services firm, attendees identified some ways that their new employee intake procedures created disparities, offering new employees from “elite” universities significant advantages over those from less “elite” universities, and immediately changed their onboarding procedure to correct this inequity. At a large law firm, attendees identified longstanding gender disparities arising from mentoring activities that involved taking junior associates to a gender-segregated spa, which enabled men, but not women, to have more face-time with the senior partners – all of whom were men. These and many other examples supplement the systemic, empirical research on the bias habit-breaking training to instill confidence that empowerment-based approaches can have widespread, positive effects within individuals and institutions.

I regularly receive emails from attendees, sometimes months or even years after they received the training, sharing how they applied the tools they learned in a way that had a major impact in their lives, sometimes even leading to large-scale changes that address some forms of institutional biases/inequities in their organization (e.g. the mail sorting example, above). The training often helps people make concerns more concrete that have previously been vague or uncertain – content from the training gives employees and employers a common language with which to discuss issues related to bias or diversity, and provides solutions for how to address those issues. For me, these anecdotal examples further drive home the importance of a DEI training being equipped to address the infinity variability of bias, as I discussed previously. As someone designing and delivering a bias training, I could never predict or anticipate all the various forms of personal and systemic biases that might be at play in someone’s life. However, if a DEI training empowers people as autonomous agents of change who look out for potential biases and are equipped with effective tools to address those biases, most people will make the most of what they learned and do the work to create a more inclusive, less biased world.

## General discussion

I would like to acknowledge that, although they are often non-scientific and often adopt information deficit model approaches, DEI trainings out in the world are predominantly developed by people who are earnestly invested in having a positive impact, and they likely draw people’s attention to factual issues related to bias and diversity. But good intentions alone do not translate to effectively creating meaningful change. We all interact with other humans on a daily basis, but that does not make us experts on human behavior any more than the blood in our arteries makes us experts on blood flow dynamics. Human behavior, like blood flow, is governed by underlying lawful processes that extend beyond simple observations. The goal of understanding, predicting, and changing human behavior is best served by the scientific method, and developing DEI trainings is no exception. If one believes in the scientific method and wants to influence human behavior, then one should look to the science of cognitive and behavioral change, and insist on evidence-based, experimentally tested approaches to DEI training.

The abundant, increasingly publicized failures of the DEI industry could lead people to feel discouraged, helpless, and defeated with regard to making positive changes related to bias, diversity, equity, and inclusion. Empowerment-based approaches, however, show considerable promise, and give reasons to hope that we can make positive changes. The bias habit-breaking training is just one initial example of the benefit of adopting an empowerment-based approach and believing in people. It has been successful, where so many other trainings have failed, because (1) the training promotes active, self-sustaining change efforts, (2) it teaches customizable, generalizable tools that equip people to address many various forms of bias, (3) it is built on a solid, scientific model of cognitive-behavioral change, and (4) rather than trying to impose change on people, it respects their autonomy and empowers them to become agents of change themselves. In short, its approach believes the best in people, and helps them to be as effective as possible at living up to their own best intentions. Whether related to DEI or any other domain of human behavior, when we want to create lasting changes, we should believe in science, believe in people, and insist on evidence-based approaches to cognitive-behavioral change.

## Figures and Tables

**Table 1.** Beck’s (2021) 10 Core Principles of CBT, and their parallels in the bias habit-breaking training

CBT...	The bias habit-breaking training...

Is based on an ever-evolving formulation of patient and their problems in cognitive terms	Teaches participants that bias unfolds in myriad ways, and gives them concrete cognitive terminology to understand various forms of bias
Requires a solid relationship between patient and therapist	Is delivered by an expert presenter, with both deep content expertise and skill developing rapport with participants
Emphasizes collaboration and active participation	Empowers participants to operate as autonomous agents of change
Is goal-oriented and problem-focused	Orients participants to specific, actionable steps they can take to make change
Initially emphasizes the present	Starts participants focusing on what they can influence most in the present, providing a foundation to build on
Is educative, teaching the client to be their own therapist and emphasizing relapse prevention	Directly teaches participants how to continue applying the cognitive-behavioral change process, sustaining it into the future
Aims to be time-limited	Is designed to give participants the fundamentals needed in a single session
Has carefully structured sessions	Has a carefully structured format designed to maximize
Teaches patients to identify, evaluate, and respond to their dysfunctional thoughts and beliefs Uses a variety of techniques to change thinking, mood, and behavior	motivation and information retention Attunes participants to the key ways cognitive biases lead to disparities and inequity, and teaches them to disrupt those biases Teaches a variety of tools and skills to reduce bias, create inclusion, and promote equity

## Footnotes

1. For the minority of people whose personal values consciously *endorse* prejudice, other kinds of approaches (e.g. strong anti-prejudice social norms) might reduce the open expression of bias. It is unlikely, however, that *any* intervention will influence these people who are highly motivated to express prejudice to become positive agents of change (Forscher et al., 2015). Luckily, most people do not fall into this category.
